# Edible *Pueraria lobata*-Derived Exosomes Promote M2 Macrophage Polarization

**DOI:** 10.3390/molecules27238184

**Published:** 2022-11-24

**Authors:** Jiaqi Wu, Xiaoyu Ma, Yu Lu, Tao Zhang, Zuoqin Du, Jin Xu, Jingcan You, Ni Chen, Xin Deng, Jianbo Wu

**Affiliations:** 1 Drug Discovery Research Center, Southwest Medical University, Luzhou 646000, China; 2 Laboratory for Cardiovascular Pharmacology, Department of Pharmacology, School of Pharmacy, Southwest Medical University, Luzhou 646000, China; 3 Key Laboratory of Medical Electrophysiology, Institute of Cardiovascular Research of Southwest Medical University, Ministry of Education, Luzhou 646000, China

**Keywords:** plant, *Pueraria lobata*, exosome, macrophage, polarization

## Abstract

*Pueraria lobata* (known as Gegen) is an edible and medicinal herb that is a nutritious medicine food homology plant in China. Previous studies indicated that *P. lobata* plays an essential role in controlling cytokines. However, the exact mechanism of the inflammation response is still unknown. In this study, we observed the uptake of *P. lobata*-derived exosomes (Exos) in isolated mouse macrophages. Our results show that *P. lobata*-derived Exos shift M1 macrophages toward the M2. These data present that *P. lobata* and puerarin might exert and enhance anti-inflammatory effects through the activation of exosomes and shifts in macrophage polarization, providing strong evidence for the application of *P. lobata* as novel an anti-inflammatory therapeutic biomaterial.

## 1. Introduction

*Pueraria lobata* (known as Gegen) is an edible and medicinal herb that is a nutritious medicine food homology plant in China. The active ingredients include flavonoids, coumarin, terpenoids, and steroids [1]. Puerarin is a flavonoid extracted from *P. lobata*, and experimental studies have exhibited various effects on treating cardiovascular diseases and cancer in vitro and in vivo. These processes are correlated with several molecular mechanisms and pathways that have a fundamental role in the regulation of apoptosis, oxidative stress, and the inflammatory response [2,3,4,5,6]. In these events, the regulation between miRNAs and target genes plays an important role in pathophysiological processes [7]. Moreover, a recent study demonstrated that puerarin might downregulate plasma TNF-α levels and improve insulin sensitivity [1], indicating that *P. lobata* could play an essential role in developing new therapeutic drugs. However, the exact cause of the inflammation response is still unknown.

Exosomes (Exos) are involved in cell-to-cell communication and contain DNA, mRNA, miRNA, proteins, lipids, and substances. Accumulating evidence has shown that multiple plant-derived exosome-like nanovesicles have exhibited potential therapeutic effects on cancer, anti-diabetic and anti-inflammatory activities, and oxidative stress in vitro and in vivo [8,9,10,11,12,13]. However, plant-derived exosomes have not been extensively studied for their ability to regulate inflammatory cells directly. Thus, it is a compelling hypothesis that *P. lobata*-derived Exos deliver functional miRNA cargoes to immune cells to regulate proinflammatory responses in vitro. Therefore, we investigated the mechanisms of *P. lobata*-derived Exos via the modulation of macrophages. We demonstrated that the *P. lobata*-derived Exos promoted M2 macrophage polarization from the induced M1 macrophage. We provide a novel concept that delivering *P. lobata*-derived Exos effectively and safely improves anti-inflammatory effects.

## 2. Methods

### 2.1. Animals

C57BL/6J mice were obtained from Chengdu Gembio Inc. (Chengdu, China). The study was conducted in accordance with the Declaration of Helsinki, and the protocol was approved by the Ethics Committee of Southwest Medical University (Project identification code: 2020YJ0340).

### 2.2. Isolation of Exosomes

Fresh leaves and stems of *P. lobata* were collected from Luzhou, located in Sichuan, China. As described previously [14], exosomes were isolated from 50 g of leaves and stems by grinding with an extractor, passing the resulting juice through filter paper, and centrifuging at 10,000× *g* for 10 min. Large debris was removed by filtering the supernatant through a 0.22 μm membrane, after which exosomes were concentrated by centrifuging the sample at 5000× *g* for 10 min at 4 °C using an Amicon Ultra-4 PL 100 KDa centrifugal filter (Merck Millipore, Darmstadt, Germany). We diluted 10 μL of the above exosome suspension with calcium and magnesium ion-free PBS buffer to 1 mL, and the particle size and concentration of the exosomes were evaluated by Nano Sight NS300.

### 2.3. Dynamic Light Scattering Instrument (DLS)

For the DLS measurement of the particle size distribution, samples were diluted to 30 μL in PBS. The exosome samples were detected by a Zeta Potential Analyzer instrument (Colloidal Dynamics, Ponte Vedra Beach, FL, USA).

### 2.4. Standard Transmission Electron Microscopy (TEM)

Collected exosomes were fixed by incubation for 1 h at room temperature with 1.25% glutaraldehyde in 0.1 mol/L phosphate buffer at a pH of 7.2, centrifuged for 10 min at 1100× *g*, and washed once in phosphate buffer. Exosomes were kept in 0.2% glutaraldehyde at 4 °C until processing for the TEM analysis of platelet morphology.

### 2.5. Exosome Uptake

To detect the direct transfer of exosomes into macrophages, *P. lobata*-derived exosomes were labelled using the PKH67 Fluorescent Cell Linker Kit (PKH67; Sigma-Aldrich, St. Louis, MO, USA) and incubated with macrophages for 24 h, and then the fluorescence signals were detected.

### 2.6. Isolation of Peritoneal Macrophages

Male C57BL/6J mice were intraperitoneally injected with thioglycollate solution (Difco Laboratories, Sparks, MD, USA). After 4 days, peritoneal cells were harvested by washing the peritoneal cavity with PBS and counted by microscopy.

### 2.7. Quantitative Real-Time PCR

Macrophages were collected and total RNA was extracted using TRIzol reagent (Invitrogen, Carlsbad, CA, USA). RNA samples were pre-treated with deoxyribonuclease I (Invitrogen Life Technologies, Carlsbad, CA, USA) and a SuperScript kit (Invitrogen Life Technologies, Carlsbad, CA, USA) was used to synthesize cDNA according to the manufacturer’s recommendations. qRT-PCR was analyzed using miScript SYBR Green PCR Kits (Qiagen, Hilden, Germany). Levels of macrophage polarization and oxidative stress marker mRNAs were determined by an ABI PRISM 7700 cycler (Applied Biosystems, Foster City, CA, USA), and 18s RNA was used as an internal control. All fold changes in gene expression were determined using the 2−ΔΔCT method. The values are presented as the mean ± SEM. All primers are listed in Table S1.

### 2.8. Statistical Analysis

Data are presented as the mean ± SEM of triplicate experiments. The significance of the differences among groups was analysed by one-way analysis of variance with a post hoc test to determine group differences in the study parameters. All analyses were performed with SPSS software (version 24.0 for Windows; Armonk, NY, USA), and a level of *p* < 0.05 was defined as indicative of statistical significance.

## 3. Results and Discussion

**Figure 1:**
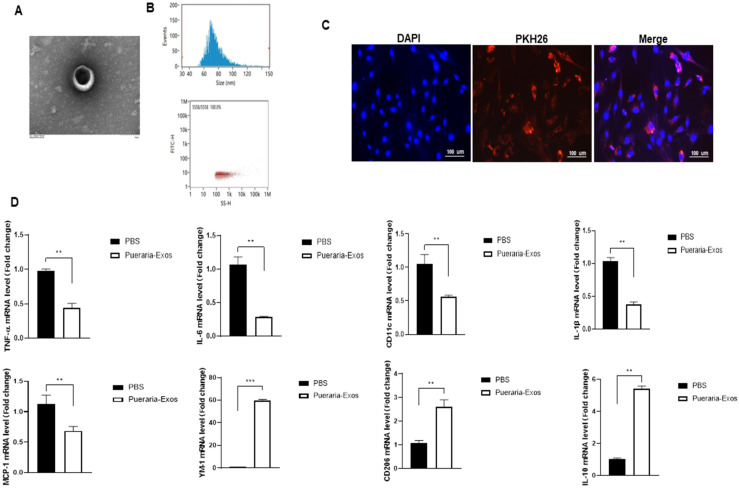
**Characterization of exosomes isolated from *P. lobata*.** (**A**) Transmission electron microscopy (TEM) images of exosomes from *P. lobata*. Scale bar, 100 nm. (**B**) Particle size distribution of *P. lobata*-derived Exos measured by dynamic light scattering. (**C**) Fluorescence microscopy images of macrophages co-cultured with PKH67-labeled *P. lobata*-derived Exos for 6 h. All scale bars = 100 μm. (**D**) The gene profiles of the M1 (IL-6, IL-1β, TNF-α, MCP-1, and CD11c) and M2 (IL-10, YM1, and CD206) phenotypes as assessed by qPCR from macrophages in vitro. Data were normalized by the amount of 18s mRNA and expressed relative to the corresponding PBS. Experiment was performed in duplicates 3 independent times. ** *p* < 0.01; *** *p* < 0.001. Data are shown as means ± SEM.

*P. lobata*-derived Exos were characterized according to TEM images and size distribution (Figure 1A,B). DLS and nanoparticle tracking analysis (NTA) were used to monitor the changes in the number and size, which remained consistent in the size distribution measurement. Exos presented a round shape with a bilayer structure (Figure 1A), and the mean diameter ranged from 40 to 150 nm (Figure 1B). These data support the successful isolation of exosomes from *P. lobata*. We further tested whether *P. lobata*-derived Exos can be taken up by mouse macrophages. This is important, as exosomes should bind with and transfer the relative molecular contents into macrophages to exert biological effects. Isolated mouse macrophages were co-cultured with culture medium containing 1.0 × 10^11^ particles/mL of *P. lobata*-derived Exos. Fluorescence microscopy images revealed that, labeled with PKH67, exosomes could be observed in the cell cytosol, and it was concluded that *P. lobata*-derived Exos can be internalized by macrophages (Figure 1C).

To validate whether *P. lobata* is associated with the macrophage phenotypic state mediated by Exos, we generated M0 macrophages (unpolarized) from normal-diet C57BJ/6-mice which were subsequently induced to the M1 (LPS + IFN-γ for 24 h) polarized state, and then the expression of macrophage polarization genes was assessed by qPCR. The results showed that the mRNA levels of pro-inflammatory genes (M1-like macrophage), including IL-6, IL-1β, TNF-α, MCP-1, and CD11c, were significantly downregulated in the treatment of *P. lobata*-derived Exos compared with PBS alone (Figure 1D). Similarly, the treatment of *P. lobata*-derived Exos upregulated the mRNA levels of IL-10, YM1, and CD206. In summary, we isolated exosomes from *P. lobata* and gave them macrophage-uptaking capability, which significantly promoted M1-to-M2 polarization.

## Data Availability

The data presented in this study are available on request from the corresponding author.

## Supplementary Materials

The following supporting information can be downloaded at: https://www.mdpi.com/article/10.3390/molecules27238184/s1. Table S1. Primers used in qPCR.
