# Supplementary material for: Edible Pueraria lobata-Derived Exosomes Promote M2 Macrophage Polarization
Source: Molecules. 2022 Nov 24;27(23):8184. doi: 10.3390/molecules27238184 (PMC9735656; doi:10.3390/molecules27238184)
Supplement: Supplementary file 1 [file molecules-27-08184-s001.zip › molecules-1967124-supplementary.pdf]

Primers used in qPCR

Gene	Forward	Reverse
CD11c	CACTCAGTGACTGCCCAAAA	CCTCAAGACAGGACATCGCT
IL-1β	ACTACAGGCTCCGAGATGAACAAC	CCCAAGGCCACAGGTATTTT
CD206	CATGGATGTTGATGGCTACTGGAG	GTCTGTTCTGACTCTGGACACTGG
MCP-1	ATGCAGGTCCCTGTCATG	GTTCACTGTCACACTGGTCA
IL-6	CACATGTTCTCTGGGAAATCG	TTGTATCTCTGGAAGTTTCAGATTGTT
TNF-α	ACGGCATGGATCTCAAAGAC	AGATAGCAAATCGGCTGACG
Ly6C	ACTGTGCCTGCAACCTTGTC	CACACAGTAGGGCCACAAGA
IL-10	TGTCAAATTCATTCATGGCCT	ATCGATTTCTCCCCTGTGAA
Ym1	AGAGTGCTGATCTCAATGTGG	GGGCACCAATTCCAGTCTTAG
18s	CCTGGATACCGCAGCTAGGA	GCGGCGCAATACGAATGCCCC
